# Mechanical Circulatory Support in Cardiovascular Surgical Patients: Single Center Practice and Experience

**DOI:** 10.31083/j.rcm2309291

**Published:** 2022-08-24

**Authors:** Xin Han, Yun-tai Yao

**Affiliations:** ^1^Department of Anesthesiology, Fuwai Hospital, National Center for Cardiovascular Diseases, Peking Union Medical College and Chinese Academy of Medical Sciences, 100037 Beijing, China; ^2^Department of Anesthesiology, Lishui People’s Hospital, The Sixth Affiliated Hospital of Wenzhou Medical University, The First Affiliated Hospital of Lishui University, 323000 Lishui, Zhejiang, China

**Keywords:** intra-aortic balloon counter pulsation, extracorporeal membrane oxygenation, ventricular assist device, indications, complications

## Abstract

**Background:**

In view of the role of mechanical circulatory support in patients with severe cardiac insufficiency during perioperative period, we searched the relevant articles on mechanical circulatory support at Fuwai Hospital, and analyzed the indications and complications of different mechanical circulatory support methods.

**Methods:**

Relevant studies were identified by computerized searches of PubMed, Ovid, Embase, Cochrane Library, Wanfang Data, VIP Data, Chinese BioMedical Literature & Retrieval System (SinoMed), and China National Knowledge Infrastructure (CNKI), using search words (“intra-aortic balloon counter pulsation” OR “IABP” OR “extracorporeal membrane oxygenation” OR “ECMO” OR “ventricular assist device” OR “VAD”) AND (“Fuwai” OR “fuwai”). All studies concerning the application of IABP, ECMO, and VAD at Fuwai Hospital were included, exclusion criteria included: (1) studies published as review, case report or abstract; (2) animal or cell studies; (3) duplicate publications; (4) studies lacking information about outcomes of interest.

**Results:**

A total of 36 literatures were selected for analysis. The specific mechanical circulatory support methods of ECMO and VAD retrieved from the studies were VA-ECMO and LVAD. The number of cases using IABP, ECMO, LVAD was 1968, 972, 67; and the survival rate was 80.4%, 54.9%, 56.7%, respectively. The major complications of IABP, ECMO and LVAD were hemorrhage (1.2%, 35.9% and 14.5%), infection (3.7%, 12.7% and 9.7%), acute kidney injury (9.1%, 29.6% and 6.5%), the secondary complications were limb ischemia, neurological events, cardiovascular events and thrombosis.

**Conclusions:**

The present study suggested that, IABP, ECMO and VAD, either alone or in combination, were effective and safe mechanical circulation support when managing cardiovascular surgical patients with severe hemodynamic instability at Fuwai Hospital.

## 1. Introduction

Approximately 3–5% cardiovascular surgical patients developed postoperative myocardial dysfunction, which require inotropic and/or vasoactive agents as first-line treatment. However, preliminary evidence suggested that administration of inotropic and/or vasoactive agents might be associated with increased morbidity and mortality [1].

Besides that, in some patients whose cardiac function are severely impaired, inotropic and vasoactive agents alone are often ineffective in maintaining stable hemodynamics, which necessitates additional mechanical circulatory support [2, 3]. Intra-aortic balloon counter pulsation (IABP), extracorporeal membrane oxygenation (ECMO) and ventricular assist device (VAD) are three most-commonly used mechanical circulatory support modalities [4]. IABP which can improve coronary perfusion pressure, is the first-line mechanical circulatory support modality for adult patients undergoing cardiovascular surgery [5]. ECMO is capable of providing circulatory and pulmonary support for patients who is refractory to conventional therapy, by means of veno-venous cannulation (V-V ECMO) or veno-arterial cannulation (V-A ECMO) [6]. VAD is reserved for patients with end-stage congestive heart failure who are refractory to conventional therapy as (1) a bridge-to-transplantation (BTT), (2) a bridge-to-recovery (BTR), (3) a bridge to decision (BTD), (4) a destination therapy (DT) [7]. For patients with different types of heart failure, VAD can be classified as left ventricular assist device (LVAD), right ventricular assist device (RVAD) and biventricular assisted device (BiVAD).

Great variance exists among different cardiovascular centers when utilizing these mechanical circulatory support modalities, due to device availability, clinical experience and cost effectiveness issues. Fuwai Hospital is the National Center for Cardiovascular Diseases (NCCD) of China, and one of the largest cardiovascular centers in the world. First clinical application of IABP, ECMO and VAD at Fuwai Hospital was in 1972 [8], 1990 [9] and 1995 [10], respectively. The present study aimed to summarize the single center experience of utilizing IABP, ECMO and VAD in cardiovascular surgical patients.

## 2. Materials and Methods

### 2.1 Search Strategy

All published clinical studies were retrieved to summarize the application of mechanical circulatory supports (IABP, ECMO and VAD) in cardiovascular surgical patients at Fuwai Hospital. All relevant publications were searched till October 1st, 2021. Relevant studies were identified by computerized searches of PubMed, Ovid, Embase, Cochrane Library, Wanfang Data, VIP Data, Chinese BioMedical Literature & Retrieval System (SinoMed), and China National Knowledge Infrastructure (CNKI), using search words (“intra-aortic balloon counter pulsation” OR “IABP” OR “extracorporeal membrane oxygenation” OR “ECMO” OR “ventricular assist device” OR “VAD”) AND (“Fuwai” OR “fuwai”).

### 2.2 Inclusion and Exclusion Criteria

All studies concerning the application of IABP, ECMO, and VAD at Fuwai Hospital were included. Primary outcomes of interest included successful weaning rate and patient survival rate, the incidence of complications/adverse effects of mechanical circulatory support (e.g., cardiovascular events, bleeding, limb ischemia, thrombosis, renal failure, neurological events and infection). Secondary outcomes of interest included anticoagulant strategy, mechanical ventilation settings, inotropic and vasoactive support requirement. Exclusion criteria included: (1) studies published as review, case report or abstract; (2) animal or cell studies; (3) duplicate publications; (4) studies lacking information about outcomes of interest. The two authors independently reviewed the titles and abstracts of all identified studies for eligibility, excluding obviously ineligible ones.

### 2.3 Data Abstraction

The following data from the included studies were abstracted to a data collection form by each author independently: (1) author, year, and journal; (2) total number of patients, sex, age, surgery; (3) anticoagulant management, respiratory management, successful weaning rate and patient survival rate after mechanical circulatory support; (4) complications/adverse events during mechanical circulatory support. Disagreements were resolved by discussion between both authors during the process of data abstraction.

## 3. Results

### 3.1 Search Results

**Fig. 1.**
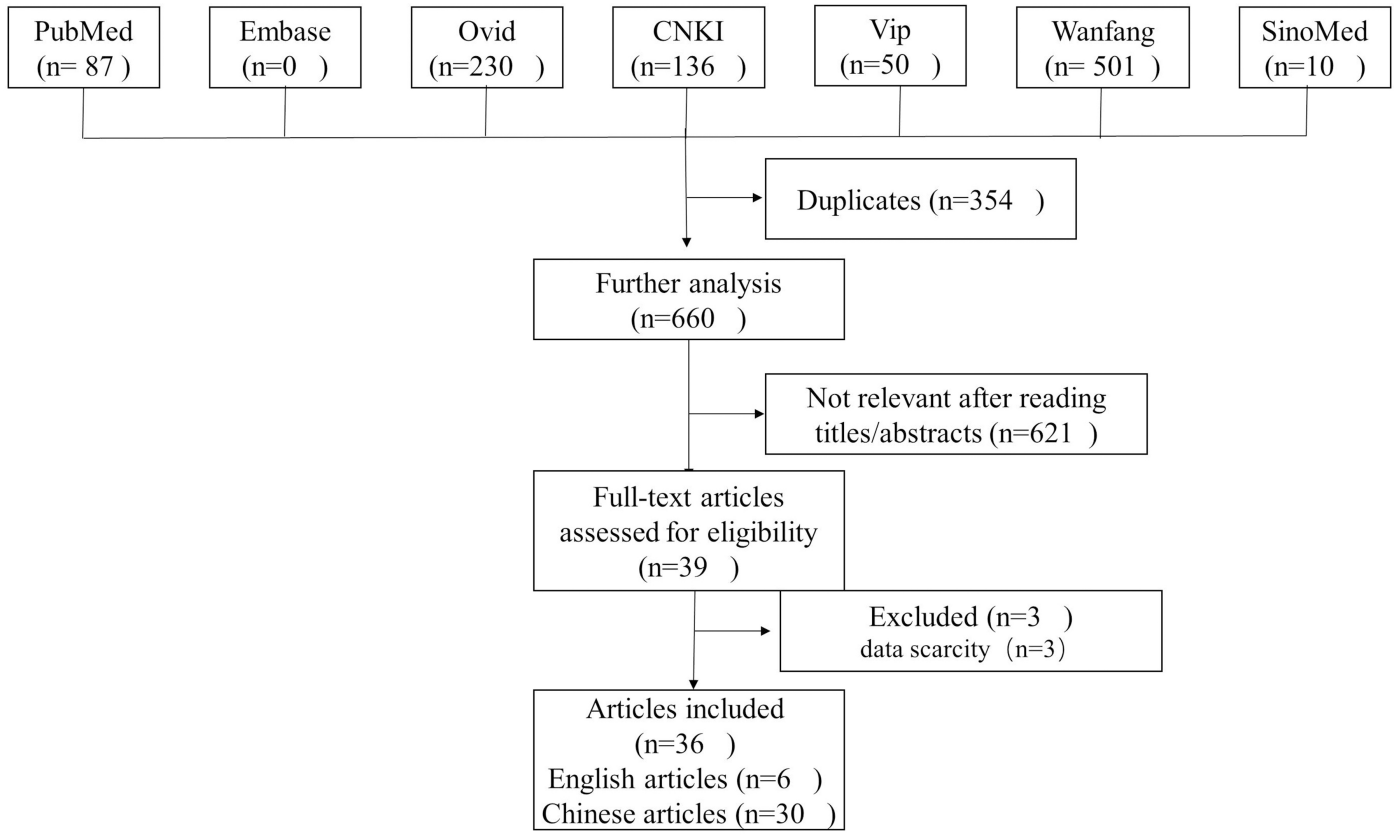
**Flow diagram of the study selection process**.

As depicted in the flowchart (Fig. 1), the initial database search identified 1014 results. Finally, thirty-six studies were determined eligible and included. Among the thirty-six studies, six were written in English and the other thirty were written in Chinese, including twenty-seven retrospective studies, seven descriptive studies and two non-randomized Controlled Trial (n-RCT) studies.

The duration of these studies followed from 1972 to 2017. Nine, fifteen and nine studies reported single application of IABP, ECMO and LVAD, respectively. The other three studies reported combined use of different mechanical circulatory support modalities.

### 3.2 Patient Characteristics

Nine included studies reporting IABP support involved 1968 patients whose ages ranged from 3.5 years to 82 years (Table 1). The proportion of male patients across the studies was 72.0% (1365/1897, patient gender was not mentioned in 71 patients).

**Table 1.** **Comparison of the characteristics of mechanical circulatory support**.

	IABP	ECMO	LVAD	ECMO+IABP
References (n)	9	15	9	3
Patients (n)	1968	972	67	145
Male/Female	1365/${\mathrm{532}}^{1}$	697/275	66/1	108/37
Age	3.5–82 years	5 days–80 years	32–71 years	—
Anticoagulants	Heparin	Heparin	Heparin/Warfarin	Heparin
Target ACT (sec)	150–180	120–200	180–200	140–180
Respiratory support mode	—	SIMV	SIMV	SIMV
Duration of therapy	Days to weeks	Days to weeks	Days to years	—
Successful weaning rate	65.4% (155/${\mathrm{237}}^{2}$)	66.7% (505/${\mathrm{757}}^{2}$)	77.6% (52/67)	—
Survival rate	80.4% (1582/1968)	54.9% (534/972)	56.7% (38/67)	—

^1^, patient gender was not mentioned in 71 patients. ^2^, number of cases associated with weaning off. IABP, intra-aortic balloon pump; ECMO, extracorporeal membrane oxygenation; LVAD, left ventricular assist device; SIMV, synchronized intermittent mandatory ventilation; INR, international normalized ratio; ACT, activated clotting time; APTT, activated partial thromboplastin time.

Fifteen studies containing 972 patients that reported were included on the use of ECMO. These studies were performed during the period from 2004 to 2017. The target population comprised pediatric and adult patients from 5 days to 80 years (Table 1). The proportion of male patients across the studies was 75.2% (697/972).

Nine studies containing 67 patients that reported were included on the use of LVAD. The duration of these studies was searched from 1995 to 2010. The target population comprised pediatric and adult patients from 32 years to 71 years (Table 1). The proportion of male patients across the studies was 98.5% (66/67).

### 3.3 Circulatory Support 

#### 3.3.1 Indications

IABP was widely used at Fuwai Hospital, indications of IABP insertion included cardiogenic shock, difficult extracorporeal circulation weaning, low cardiac output syndrome, intractable cardiac arrhythmia and bridge to other mechanical circulatory support, *etc*.

ECMO was regularly used at Fuwai Hospital for patients with acute cardiogenic shock. The implantation of ECMO was mainly used to maintain the hemodynamic stabilization, so VA-ECMO was routinely used. The discussion of ECMO in this study also revolved around VA-ECMO.

The main indication of VAD surgical techniques in the treatment of heart failure at Fuwai Hospital included: (1) bridge to transplantation; (2) bridge to recovery; (3) bridge to decision and destination therapy in transplant-ineligible patients. Right ventricular assist device (RVAD) and biventricular assisted device (BiVAD) were not widely performed at Fuwai Hospital. This was different from the use in other countries, which may be associated with the cultural factors and economic conditions of Chinese patients. Because of this, LVAD was used for analysis in this study.

#### 3.3.2 Operating Time

Of the 1968 IABP-supported patients, IABP was installed in 370 (18.8%) patients preoperatively, 602 (30.6%) patients intraoperatively and 857 (43.5%) patients postoperatively (Table 1). Operating time was not mentioned in the other 139 (7.1%) patients.

Of the 972 ECMO-supported patients, ECMO was installed in 3 (0.3%) patients preoperatively, 886 (91.2%) patients intraoperatively and 83 (8.5%) patients postoperatively (Table 1).

Of the 67 LVAD-supported patients, LVAD was installed in 5 (7.5%) patients preoperatively and 62 (92.5%) patients intraoperatively (Table 1).

#### 3.3.3 Anticoagulant Strategy

Activated clotting time (ACT) was routinely used to monitor heparinization and its reversal by protamine at Fuwai Hospital. Activated partial thromboplastin time (APTT) test was used for evaluation of low dose heparinization.

During the IABP-supported period, only 1 article described the anticoagulation with heparin to maintain ACT in the range of 150 seconds to 180 seconds (Table 1), the other studies did not elaborate on anticoagulation. Besides, at Fuwai Hospital, there was no significant difference in the incidence of limb ischemia and bleeding when the IABP was employed.

During the ECMO-supported period, all patients were treated in the same way during the procedure with commencement of anticoagulation with heparin until ACT in the range of 120 seconds to 200 seconds and APTT in the range of 40 seconds to 70 seconds (Table 1). We infused platelet and leukocyte-reduced packed red blood cells to maintain platelet counts more than 50 × ${\mathrm{10}}^{9}$/L and hematocrit between 30% to 35%.

During the LVAD-supported period, patients were treated with heparin to maintain the ACT in the range of 180 seconds to 200 seconds (Table 1). However, when the patients were awake and their gastronintestinal function recoverd, warfarin could be used to maintain INR 2–3.

#### 3.3.4 Inotropic and Vasoactive Support

At Fuwai Hospital, inotropes for continuous intravenous infusion included dopamine, dobutamine, milrinone, epinephrine, levosimendan, *etc*. Vasopressors mainly included norepinephrine, methoxamine, vasopressin, and vasodilators included nitroglycerin, isosorbide dinitrate, nitroprusside, nicardipine, urapidil. During the mechanical circulatory support period, vasoactive drugs were administrated to minimum for cardiac rest. This was also applicable in pediatric patients. If the condition of patients improved to a satisfactory state, the flows of mechanical circulatory support were gradually reduced at hourly intervals during a 12–48 hours period, the inotropic drugs were increased at the same time according to the hemodynamic monitor, and the patient was weaned off if the circulation was stable. During the IABP-supported period,the use rates of nitrates and inotropic drugs were 24.7% and 9.3% respectively. The rates of nitrates and inotropic drugs were not mentioned in other literatures.

#### 3.3.5 Mechanical Ventilation

There was no difference in respiratory management among IABP, ECMO and LVAD. Synchronized intermittent mandatory ventilation (SIMV) mode was applied to all patients when mechanical ventilation was used. The ventilator was maintained: tidal volume was 8–10 mL/kg; respiratory rate was 10–30 breaths per minute; fraction of inspired oxygen (${\mathrm{FiO}}_{2}$) was 0.30–0.60; peak end-expiratory pressure (PEEP) was between 4 to 9 cm $H_{2}$O; The cardiac and pneumonic functions were assessed by the result of echocardiograph, hemodynamics and blood gas anslysis, adjusting the indicators of the ventilator according to the feedback.

#### 3.3.6 Weaning of Circulatory Support

There was no unified standard for IABP weaning. At Fuwai Hospital, when the patients’ conditions significantly improved, the inotropic drugs dose was gradually reduced, the hemodynamics was stable, and the IABP could be weaned off. The average support time of IABP was between 109 hours and 131 hours.

If the condition of patients improved to a satisfactory state, the ECMO flows would be gradually reduced to 10% of the patient’s cardiac output, and the inotropic drugs were gradually adjusted according to the hemodynamic monitor, weaning could be considered. The average support time of ECMO was between 108 hours and 128 hours.

If the patients’ hemodynamic stability or received a heart transplant, the LVAD could be weaned off. The support time of LVAD ranged from 9.5 hours to 2 years.

#### 3.3.7 Complications and Survival Rate

At Fuwai Hospital, the most common complications after IABP therapy were acute kidney injury (9.1%), neurological complications (5.5%), infection (3.7%), hemorrhage (1.2%), cardiovascular events (1.2%), and limb ischemia (1.2%) (Table 2). The figure was even higher for critically ill patients, such as patients with advanced age, diabetic mellitus, or concomitant ECMO support was independent risk factor of complications associated with IABP. Prophylactic using of IABP of preoperative could reduce the incidence of complications. The survival rate of IABP-supported patients was 80.4% (1582/1968 patients) at Fuwai Hospital (Table 1), and patients who underwent coronary artery bypass grafting with IABP had a survival rate 95.9%.

**Table 2.** **Comparison of the complications of mechanical circulatory support**.

Complications (n)	IABP	ECMO	LVAD	ECMO+IABP
Acute kidney injury	9.1% (180/1968)	29.6% (248/${\mathrm{839}}^{1}$)	6.5% (4/${\mathrm{62}}^{1}$)	34.5% (50/145)
Hemorrhage	1.2% (24/1968)	35.9% (256/${\mathrm{713}}^{1}$)	14.5% (9/${\mathrm{62}}^{1}$)	29.7% (43/145)
Infection	3.7% (73/1968)	12.7% (91/${\mathrm{713}}^{1}$)	9.7% (6/${\mathrm{62}}^{1}$)	9.0% (13/145)
Limb ischemia	1.2% (24/1968)	12.2% (87/${\mathrm{713}}^{1}$)	—	21.4% (31/145)
Neurological events	5.5% (109/1968)	11.2% (80/${\mathrm{713}}^{1}$)	6.5% (4/${\mathrm{62}}^{1}$)	13.8% (20/145)
Cardiovascular events	1.2% (24/1968)	5.7% (41/${\mathrm{713}}^{1}$)	11.3% (7/${\mathrm{62}}^{1}$)	—
Thrombosis	—	10.1% (72/${\mathrm{713}}^{1}$)	1.6% (1/${\mathrm{62}}^{1}$)	13.8% (20/145)

^1^, number of cases associated with complications. IABP, intra-aortic balloon pump; ECMO, extracorporeal membrane oxygenation; LVAD, left ventricular assist device.

The complications during the period of ECMO included renal failure (29.6%), access-site or gastrointestinal hemorrhage (35.9%), infection (12.7%), limb ischemia (12.2%), neurological complications (11.2%), multiple organ dysfunction syndromes during hospitalization (10.8%) and thrombosis (10.1%) (Table 2). The overall weaning rate of ECMO-supported patients was 66.7% (505/757 patients) and survival rate was 54.9% (534/972 patients) at Fuwai Hospital (Table 1).

The most frequent postoperative complications of LVAD were hemorrhage (14.5%), infection (9.7%), acute kidney injury (6.5%), neurological complications (6.5%) and cardiovascular events (11.3%) (Table 2). The overall weaning rate of LVAD-supported patients was 77.6% (52/67 patients) and survival rate was 56.7% (38/67 patients) at Fuwai Hospital (Table 1). The articles published by Fuwai Hospital on the use of LVAD were concentrated before 2010.

## 4. Discussion

Patients with cardiogenic shock are conservatively managed using high-dose vasoactive medications for hemodynamic support [11]. However, there are still a large number of patients who die due to circulatory collapse. Mechanical circulatory support had become a necessary means of life support [12]. In recent years, the utilization rate of mechanical circulatory support is increasing. About 3%–8% of the patients undergoing cardiovascular surgery require mechanical circulatory assist devices.

IABP is the most widely used mechanical circulatory support device. IABP can reduce left ventricular afterload and increase coronary artery blood flow, so it is widely used in low cardiac output and failure of weaning off CPB patients. Due to its low complication rate, fast manner of insertion, convenience and lower medical costs, when the patient exceeded the limit dose of vasoactive drugs and had the indications for IABP, the attending physician would be highly motivated to use IABP.

However, IABP with such an excellent advantages also has its limitations. Over the past decade, the appropriate using of IABP has been subject to hevey controversy, the focuses on the automatic regulation of coronary blood flow [13]. Autoregulation of coronary blood flow is based on the premise that myocardial metabolism remains constant. Under normal physiologic circumstances, myocardial blood flow remains arterioles constrict or dilate over a wide range of aortic pressures 45–120 mmHg [14]. This is known as coronary autoregulation. For coronary arteries with automatic adjustment function, IABP implantation is of little significance. Therefore, when IABP was inserted, we should choose the condition of coronary artery autoregulation failure. When coronary blood flow directly depends on perfusion pressure, IABP can improve coronary blood flow [15]. This can only be expected with exhausted coronary autoregulation, typical in acute myocardial infarction complicated by persistent ischemia. In this situation, augmented diastolic pressure was expected to increase myocardial oxygenation [16]. The concept was further corroborated by a sub-study of the CRISP AMI study. Retrospective studies had shown that patients with large ST-elevation myocardial infarction (STEMI) who were treated with IABP had significantly improved survival rate [17, 18]. In persistent ischemia situation, myocardial blood flow was proportional to perfusion pressure. The inflation of IABP during diastole could increase coronary artery perfusion pressure, thereby increasing coronary blood flow and increasing myocardial oxygen supply, which was of great help to the recovery of damaged myocardium [19, 20].

Should IABP be implanted in all patients with cardiogenic shock? The IABP-SHOCK II Trial showed that there was no significant difference in short-term and long-term survival of patients with cardiogenic shock complicating acute myocardial infarction with or without IABP implantation [21, 22]. Thanks to these studies, IABP had become a lower priority, avoiding unnecessary complications and reducing the financial burden on patients. We also believe that IABP was not a routine treatment for all patients with cardiogen Mechanical circulatory support should be considered only if medication and other treatments were difficult to maintain hemodynamic stabilization. This required clinicians to have rich clinical experience, strictly control indications, weigh the advantages and disadvantages of all parties in the face of critically ill patients, and finally formed the most beneficial treatment plan for patients.

In recent years, with the development of cardiovascular surgery, the use of ECMO was also increasing. ECMO played an important role in the treatment of severe cardiopulmonary failure, malignant arrhythmia, postoperative refractory low cardiac output, and failure to escape from cardiopulmonary bypass [23, 24, 25]. ECMO can also be an important adjunctive tool in the management of patients awaiting heart transplantation [26, 27]. In addition, ECMO is often used as an adjunct in cardiopulmonary resuscitation and high-risk coronary interventions, making it an attractive first choice in refractory cardiogenic shock [28]. Although ECMO provides circulatory support with rapid application, it causes an addition increases in afterload and decreases the blood flow in coronary arteries due to retrograde blood flow, which potentially deteriorates cardiac function, increases pulmonary congestion, and an increased need for vasoactive medications.

ECMO was regularly used at Fuwai Hospital for patients with acute cardiogenic shock, The utilization rate of ECMO was increased year by year. However, under the circumstance of COVID-19, the surgical volume and the patients using ECMO decreased in 2020. This could be known form the annual outcomes of cardiovascular surgery of Fuwai Hospital [29]. ECMO and IABP used in combination was routinely used for short-term ventricular assistance. In 2019, the combined use of the two accounted for 54.5% of the total Both applications have achieved excellent outcomes [30]. This result was consistent with Professor Berg’s research [31].

Given the increasing incidence of end stage heart failure and the persistently inadequate supply of donor organs, LVAD was developed to support patients on the wait-list for heart transplantation [32]. LVAD is an important life support equipment, it could prolong the patient’s life and Wait for further treatment. LVAD technology continues improve, as a result, more patients with LVADs could wait longer until getting orthotopic heart transplantation [33]. In the United States, the annual use of LVAD devices had exceeded 2500 annually [34, 35]. The third generation of VAD, featuring magnetic levitation and contactless bearings, is one of the most advanced artificial hearts in the world. CH-VAD was the first-generation of magnetic levitation blood pump with completely independent intellectual property rights in China. It was launched by Professor Shengshou Hu and his artificial heart team in June 2017. By the end of 2020, a total of 42 patients were implanted with LVAD, 40 patients were discharged alive with LVAD device, and 2 patients died perioperative. The device was withdrawn in 1 patient with cardiac function recovery, 3 patients received heart transplantation, the remaining 38 patients were follow up for 363–1073 days. One of these patients had been living with the device for more than 40 months and was the longest in China. The 1-year and 2-year survival rates were 100% and 85%, respectively. According to current clinical results, in addition to heart transplantation, LVAD implantation is gradually becoming the most effective surgical treatment for end-stage heart failure in China [29]. Although LVADs offered a way for patients with end-stage heart disease to extend life, patients with severe comorbidities and some specific cardiomyopathies may be not suitable to implante LVAD, such as dilated cardiomyopathy secondary to KSS, thasthyretin amyloidosis, *etc*. [36, 37, 38, 39]. In addition, low body surface area, prior aortic valve replacement, coagulopathy, primary right ventricular dysfunction, and sociocultural issues were important factors limiting the use of LAVD [40].

Mechanical circulation support saved people’s lives, while there were also many complications threatening patients’ survival, among which the three most important complications are acute renal failure, bleeding, and infection [41, 42].

Acute kidney injury (AKI) was a common complication after cardiac surgery, with an incidence between 7% and 40% had been reported depending on the definition used [43]. At Fuwai Hospital, the incidence of AKI in patients was 9.1% with IABP implantation and 6.5% with LVAD implantation. Compared to the use of IABP, the use of ECMO had higher incidence of complications. The incidence of AKI was 29.6% in patients treated with ECMO and 34.5% in patients treated with ECMO+IABP. This was an interesting result. Studies had confirmed that compared with continuous flow, pulsatile blood flow was more beneficial to the viscera perfusion [44]. IABP implantation could increase coronary blood flow, reduce myocardial afterload, stabilize hemodynamics, and decrease the use of vasoactive drugs [45], so as to increase renal perfusion and reduce renal toxicity caused by drugs. Patients with LVAD implantation also had a lower incidence of AKI, which may be related to LVAD placement providing better circulation support and ensuring organ perfusion, but the data bias caused by sample size cannot be excluded.

In this study, it was found that ECMO and ECMO+IABP groups had the highest incidence of AKI, which was largely related to the baseline characteristics of patients using ECMO. Poor basic conditions of patients may be one of the reasons for the high incidence of kidney injury. At Fuwai Hospital, ECMO was mainly used to treat patients with acute cardiogenic shock, and postoperative users accounted for 65.9% (29/44) [30]. Impaired cardiac output directly affected renal perfusion, and even with the assistance of ECMO, the incidence of acute kidney injury also was high.

Both coagulant factors and anticoagulant factors were activated when mechanical circulation support was implanted. “Thrombus or hemorrhage?” was a lot depends on the relative balance of the two. The embolization was the result of an abiotic device in contact with the blood. ECMO was a cardiopulmonary bypass device, when blood came into contact with the tube, coagulation factors and platelets were activated and thrombus was easily formed. Therefore, anticoagulant therapy was required if the ECMO was running. At Fuwai Hospital, the anticoagulant measures implemented in ECMO were mainly intravenous injection of ordinary heparin and maintained the ACT 120–200 s. The incidence of bleeding complications in ECMO patients was 35.9%. Bleeding was one of the most common complications in the use of ECMO. In the 2016 Extracorporeal Life Support Organization (ELSO) registry report, the incidence of bleeding with VA-ECMO described was 44% in adult [46] The high bleeding rate of ECMO was closely related to the use of anticoagulant drugs and the stress response. In addition, ECMO was often used in critically ill or post-operative patients, which were high risk factors for bleeding complications.

Hemorrhage complications also represented the most common cause of hospital admission during the course of LVAD patients. Mucosal bleeding was a frequent and morbid complication observed in 20% to 81% of patients depending on age and presence of other important risk factors [33, 47]. At Fuwai Hospital, the bleeding complications of LVAD patients observed in 14.5%. In the European TRACE study, the authors concluded that avoiding antiplatelet therapy might have lowered the risk of bleeding, whilst maintaining stroke and pump thrombosis rates similar to previous trials [48].

Infections were also common complications of mechanical circulatory support therapy. The incidence of using IABP, ECMO and LVAD was 3.7%, 12.7% and 9.7%, respectively. In a study of 74 patients who received LVAD implants after heart transplantation from 2008 to 2017, in Department of Medicine, Stanford University, twenty-one patients (28.3%) developed an infection while supported by an LVAD [49]. A recent analysis by the International Society of Heart and Lung Transplantation Registry for Mechanically Assisted Circulatory Support also showed that infection was the most common complication (37%) [50]. Until 2010, there were 6 cases (9.7%) developed infection while using LVAD at Fuwai Hospital. In the 42 patients implanted with CH-VAD between 2017 and 2020, the incidence of infections were 13%, lower than the reported [30]. Compared with IABP and ECMO, more attention had been paid to LVAD-induced infections. That was because that the use of LVAD was closely related to heart transplantation. Professor Sohail and his team found that patients with device infection before transplantation did increase the postoperative recovery time compared with those without infection, but no significant difference was observed in the survival after transplantation. Therefore, when severe or refractory LVAD device infection cannot be cured, infection suppression can be selected to wait for the donor [51].

In China, although the basic social medical insurance can reimburse part of the cost, mechanical assistance is still too expensive for most patients. The high cost limits the use of mechanical assistance facilities, and various complications during the use of mechanical assistance undoubtedly aggravate the financial burden of patients. The decision should consider the balance of cost, risk and benefit, thefore, all kinds of mechanical assistance support complications and the use of safety research are very beneficial.

## 5. Conclusions

The present study suggested that, IABP, ECMO and VAD, either alone or in combination, are effective and safe mechanical circulation support when managing cardiovascular surgical patients with severe hemodynamic instability at Fuwai Hospital.
